# Analysis of 90 Mb of the potato genome reveals conservation of gene structures and order with tomato but divergence in repetitive sequence composition

**DOI:** 10.1186/1471-2164-9-286

**Published:** 2008-06-13

**Authors:** Wei Zhu, Shu Ouyang, Marina Iovene, Kimberly O'Brien, Hue Vuong, Jiming Jiang, C Robin Buell

**Affiliations:** 1 J. Craig Venter Institute, 9704 Medical Center Drive, Rockville, MD 20850, USA; 2 Department of Horticulture, University of Wisconsin-Madison, Madison, WI 53706, USA; 3 Department of Plant Biology, Michigan State University, 166 Plant Biology Building, East Lansing, MI 48824, USA; 4 Translational Sciences, MedImmune, Inc., One MedImmune Way, Gaithersburg, MD, 20878, USA; 5 Institute for Genome Sciences, University of Maryland School of Medicine, 20 Penn Street HSF-II, Baltimore, MD 21201, USA

## Abstract

**Background:**

The Solanaceae family contains a number of important crop species including potato (*Solanum tuberosum*) which is grown for its underground storage organ known as a tuber. Albeit the 4^th ^most important food crop in the world, other than a collection of ~220,000 Expressed Sequence Tags, limited genomic sequence information is currently available for potato and advances in potato yield and nutrition content would be greatly assisted through access to a complete genome sequence. While morphologically diverse, Solanaceae species such as potato, tomato, pepper, and eggplant share not only genes but also gene order thereby permitting highly informative comparative genomic analyses.

**Results:**

In this study, we report on analysis 89.9 Mb of potato genomic sequence representing 10.2% of the genome generated through end sequencing of a potato bacterial artificial chromosome (BAC) clone library (87 Mb) and sequencing of 22 potato BAC clones (2.9 Mb). The GC content of potato is very similar to *Solanum lycopersicon *(tomato) and other dicotyledonous species yet distinct from the monocotyledonous grass species, *Oryza sativa*. Parallel analyses of repetitive sequences in potato and tomato revealed substantial differences in their abundance, 34.2% in potato versus 46.3% in tomato, which is consistent with the increased genome size per haploid genome of these two *Solanum *species. Specific classes and types of repetitive sequences were also differentially represented between these two species including a telomeric-related repetitive sequence, ribosomal DNA, and a number of unclassified repetitive sequences. Comparative analyses between tomato and potato at the gene level revealed a high level of conservation of gene content, genic feature, and gene order although discordances in synteny were observed.

**Conclusion:**

Genomic level analyses of potato and tomato confirm that gene sequence and gene order are conserved between these solanaceous species and that this conservation can be leveraged in genomic applications including cross-species annotation and genome sequencing initiatives. While tomato and potato share genic features, they differ in their repetitive sequence content and composition suggesting that repetitive sequences may have a more significant role in shaping speciation than previously reported.

## Background

The potato (*Solanum tuberosum*) tuber is a specialized underground storage organ that develops from modified stems termed stolons. Although the tuber is primarily composed of starch, it also contains high levels of proteins and due to its importance as a food source, a prime focus in potato research has been tuber quality [1-6]. Another key focus in potato research is disease resistance as potato is susceptible to several pathogens including *Phytophthora infestans*, the causal agent of late blight of potato. Molecular and genomic approaches, coupled with initial genetic mapping data, have identified resistance genes in potato against this pathogen [7-11] including a potentially viable commercial form of resistance to late blight conferred by the *RB *gene identified in the wild potato species, *Solanum bulbocastanum*, which can confer resistance to a wide range of *P. infestans *isolates[10].

Genomic resources for potato have been developed including Expressed Sequence Tag (ESTs; [12-14]), bacterial artificial chromosome (BAC) clone libraries [15,16], microarray platforms [2,17], and a dense genetic map [18]. These resources have been utilized in studies on potato physiology, development, responses to abiotic and biotic stress, polyploidy, comparative genomics as well as enhancement of genetic maps [2,17,19-26]. The potato genome is reported to be 798–931 Mb [27] and with the availability of improved sequencing technologies, coupled with decreased fiscal constraints on genome sequencing, an international consortium to sequence the potato genome has been established [28]. The Potato Genome Sequencing Consortium (PGSC) is focused on generating an initial draft sequence of the potato genome using a BAC-by-BAC approach followed by a finishing phase. The PGSC is enabled by the availability of two resources, a dense genetic map for potato [18] and an anchored Amplified Fragment Length Polymorphism-fingerprinted BAC library [28].

Collectively, the Solanaceae family is one of the world's most important vegetable families as species are grown for their tubers (potato), fruits (tomato, pepper, eggplant), leaves (tobacco), and ornamental features (petunia, *Nicotiana *species). In 2006 in the U.S., potato production was valued at $3.2 billion with tomato, tobacco, and pepper production valued at $1.6 billion, $1.2 billion, and $686 million, respectively [29]. While the cultivated species have been bred for these diverse agronomic traits, genome sequence analysis has indicated that these species share to a large extent not only genes [30] but also gene order (synteny) between their genomes [31-35]. While major classes of repetitive sequences are conserved among some Solanaceae species [36,37], lineage-specific repetitive sequences have been reported, suggesting divergence of this fraction of the genome has occurred through evolution [36-42]. With the availability of large genomic datasets for two Solanaceae species, tomato and potato, the extent of sequence conservation as well as synteny can be addressed in a more robust manner. In this study, we report on the generation of the first large set of genomic sequences from the potato genome along with characterization of these sequences with respect not only to the potato genome landscape but also in a comparative manner with genome sequences from tomato. We further compared our potato genome sequences with sequences from the collective Solanaceae transcriptome to determine the extent to which available solanaceous sequences can be used to cross-annotate the potato genome.

## Results and Discussion

### Characteristics of the potato genome

A total of 77,568 potato BACs were end sequenced from both ends resulting in 155,130 total sequences. For low quality and vector sequences, 140,259 sequences were generated with an average read length (after trimming) of 621 nucleotides representing a total of 87.14 Mb of potato genome sequence (Table 1). The average GC content of the potato BAC end sequence (BES) dataset was 35.6%, comparable to that of tomato BES dataset (36.2%) and the whole genome sequences of Arabidopsis (36.0%; [43]), poplar (33.7%; [44]), and grape (34.6%; [45] (Table 1)). Not surprisingly, the potato genome GC content was substantially lower than that of the rice genome (43.6%; [46]), a model monocotyledonous species. Using sequence derived from BACs, not BAC end sequences, the GC content of the potato and tomato genome was 34.2% and 33.6%, respectively, slightly lower yet consistent with data generated from the substantially larger BES dataset. With respect to coding potential, using the program GMAP [47], 5.5% (7,650/140,259) of the potato BES had a spliced alignment with clustered assemblies of Solanaceae ESTs, mRNAs, and cDNAs, lower albeit comparable to that observed with the tomato BES dataset (11,487/305,429 = 3.8%). The amount of sequence covered by these transcript alignments is 3.24 Mb and 5.03 Mb within the potato and tomato BES datasets, respectively.

**Table 1:** Statistics of BAC end sequence data in comparison to that of complete plant genomes.

	Potato BES	Tomato BES	Poplar	Grape	Arabidopsis	Rice
No. Sequences	140,259	305,429	19	19	5	12
Total Length	87 Mb	274 Mb	486 Mb	498 Mb	119 Mb	376 Mb
Average Length (nucleotides)	621	897	N.A.	N.A.	N.A.	N.A.
GC%	35.6	36.2	33.7	34.6	36	43.6
Repeat%	34.2	46.3	42.0^1^	41.4^2^	14^3^	34.8^4^
EST Hit	7,650 (5.5%)	11,487 (3.8%)	N.A.	N.A.	N.A.	N.A.

1. [44]

2. [45]

3 [43]; this represents only the transposable elements

4. [46]

Two sets of potato BACs were targeted for sequencing, BACs anchored on chromosome 6 and BACs putatively syntenic with tomato (Additional Data Files 1 and 2). A total of 22 potato BACs were sequenced in this study and we were able to generate 13 potato BACs in phase 2 and 3 which have ordered, oriented contigs allowing for gene annotation. Additionally, five complete (phase 3) potato BACs available in Genbank from other *S. tuberosum *BAC libraries were included in this study.

**Figure 1:**
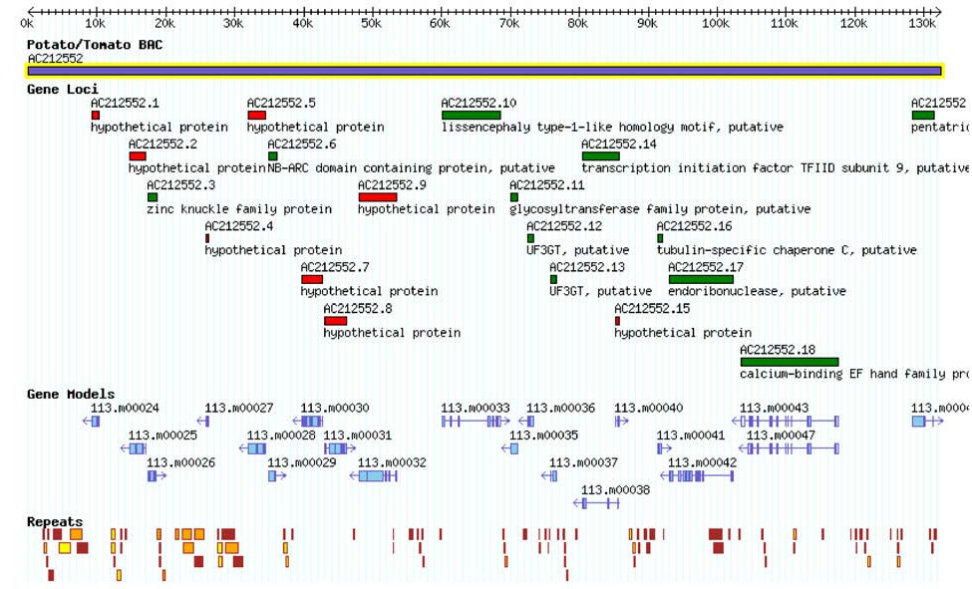
**Gene and repetitive sequence content in the candidate syntenic potato AC212552**. Shown in the figure is ~130 kb of sequence with loci, gene models, and repetitive sequences annotated. For the Loci track, genes encoding hypothetical proteins are colored in red, genes encoding expressed proteins in yellow, and genes encoding known/putative proteins colored in green. Gene models were generated using the annotation pipeline described in the Materials. Repetitive sequences are shown on the bottom track with retrotransposons colored brown, transposons colored orange, miniature inverted-repeat transposable elements colored green, centromeric-related sequences colored blue, telomeric-related sequences colored purple, rDNA sequences colored pink, and unclassified repetitive sequences colored yellow.

**Figure 2:**
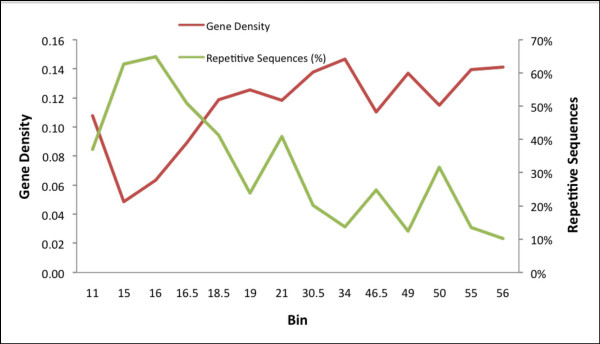
**Distribution of gene density and repetitive sequences along potato chromosome 6**. Bins are plotted on the x-axis as reported by van Os [18] with gene density (gene per kb) and repetitive sequences plotted on the y-axes. For BACs reported in a bin range, the average of the distance between the two bins was used. The centromere is located near bins 16–17 (Iovene and Jiang, unpub.).

Of the total 18 BACs in phase 2 and 3, seven BACs were selected randomly from the potato genome including chromosome 6. The other 11 BACs were identified as putatively syntenic with tomato contigs generated from the Tomato Genome Initiative (Additional Data File 2). Genes were annotated on all of the potato BACs using a semi-automated annotation pipeline; a total of 287 genes were annotated within the 18 potato BACs (Figure 1). The numbers of genes annotated as encoding "known"/"putative", "expressed," and "hypothetical" proteins are 160, 21, and 106, respectively. Using the same annotation pipeline, 221 genes (139 known/putative, 17 expressed, and 65 hypothetical genes) were annotated within the tomato BACs/contigs. Overall, the length of genes, exons, and introns of syntenic potato BACs were similar to that observed in the syntenic tomato BACs/contigs (Table 2). While GC content and exon/intron length were similar between syntenic tomato/potato BACs and randomly selected potato BACs, the average gene in randomly selected BACs had one less exon per gene and consequently were shorter (2.4 kb vs 3.1 kb). The Tomato Genome Initiative [48] is focused on the euchromatic region of the tomato genome which is highly enriched in genes in comparison to the whole genome. As a consequence, syntenic potato/tomato BACs have an increased gene density relative to random BACs. Even with data from a limited number of BACs sampled, this skew in gene density and repetitive sequence content is discernible and is illustrated on potato chromosome 6 in Figure 2. The BACs from the euchromatic arms show a higher gene density and a lower repetitive sequence content compared to those BACs in the heterochromatin containing centromeric region estimated to be at bin 16–17 (Iovene and Jiang, unpubl.)

**Table 2:** Features of potato and tomato BAC sequences.

Feature	Potato Syntenic BACs	Tomato Syntenic BACs	Random Potato BACs
Exons per gene	5.1	5.1	4.2
			
Exon length(bp)^1^	266 (18.9%)	279 (18.6%)	222 (10.9%)
Intron length(bp)^1^	441 (25.2%)	408 (21.8%)	457 (17.0%)
Gene length(bp)^1^	3,148 (44.1%)	3,086 (40.4%)	2,401 (27.9%)
			
Exon GC content	42%	42%	42%
Intron GC content	34%	34%	33%
Gene GC content	38%	38%	38%
CDS/ORF GC content	43%	43%	42%
			
First position GC	50%	49%	49%
Second position GC	40%	41%	41%
Third position GC	38%	39%	38%

^1 ^Percentages within the sequences analyzed are enclosed in the brackets.

### Sequence level conservation within the Solanaceae and its use in annotation of the potato genome

**Figure 3:**
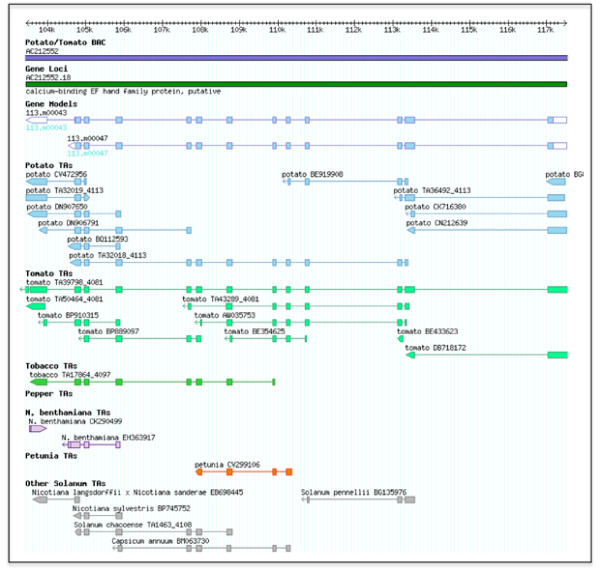
**Alignment of a selected potato gene model with transcripts from diverse solanaceous species**. Transcript Assemblies (TAs) from solanaceous species are shown aligned to the potato genome.

Representation of the respective transcriptome is variable among the set of 13 Solanaceae Transcript Assemblies used in this study [49]; sequences (Transcript Assemblies and singletons) ranged from 716 in the *S. lycopersicum *× *S. pimpinellifolium *Transcript Assembly to 81,072 sequences in the *S. tuberosum *(potato) Transcript Assembly (Additional Data File 3). Of the combined 251,274 Solanaceae Transcript assemblies and singletons, over half of the sequences are derived from potato or tomato reflecting the emphasis in EST sequencing for these two crop species. Within the 287 potato genes annotated in this study, 192 genes (129 known/putative, 21 expressed, and 42 hypothetical) have at least one supporting Solanaceae transcript (Table 3). Excluding the potato Transcript Assemblies, 153 potato genes (112 known/putative, 17 expressed and 24 hypothetical) have transcript support from other Solanaceae species demonstrating the breadth of transcript support available within the Solanaceae (Table 3). Moreover, 71 potato genes have support from at least three other Solanaceae species and as shown in Figure 3, the selected gene model (AC212552.18) is supported by transcripts from 10 different solanaceous species. Based on sequence similarity to annotated proteins, this gene model encodes a putative calcium-binding EF hand family protein. Clearly, annotation of the potato genome can be greatly enhanced by inclusion of not only cognate *S. tuberosum *transcripts, but also transcripts from other solanaceous species.

**Table 3:** Extent of transcript support for annotated potato genes among the Solanaceae transcriptome dataset.

No. Species^a^	No. Loci	
	
	Including Potato TA^b^	Excluding Potato TA^c^
1	63	50
2	36	32
3	26	31
4	28	19
5	18	11
6	11	6
7	6	3
8	3	0
9	0	1
10	1	0

Total	192	153

^a ^Potato genes (loci) were searched against 13 Solanaceae Transcript Assemblies (TA, see Additional Data File 3) datasets as described in the Materials and Methods.

^b ^The number of loci that aligned with TAs from 1–10 different Solanaceae species including the Potato TA dataset is reported.

^c ^The number of loci that aligned with TAs from 1–10 different Solanaceae species excluding the Potato TA dataset is reported.

### Synteny between potato and tomato

Previous studies with the Solanaceae [31,33,34,50,51] identified synteny between a number of Solanaceae species including potato and tomato. These studies utilized genetic markers and showed, albeit at a low resolution, conservation of gene order between potato and tomato. With the pending availability of the tomato genome sequence, we were interested in determining the extent of synteny between tomato and potato to assess whether tomato genome sequences can be used 1) to identify syntenic potato BACs for the potato genome sequencing initiative, 2) to provide contig order and orientation information for potato BACs sequenced to draft level, and 3) to provide as a "reference genome" for structural annotation of the potato genome.

**Figure 4:**
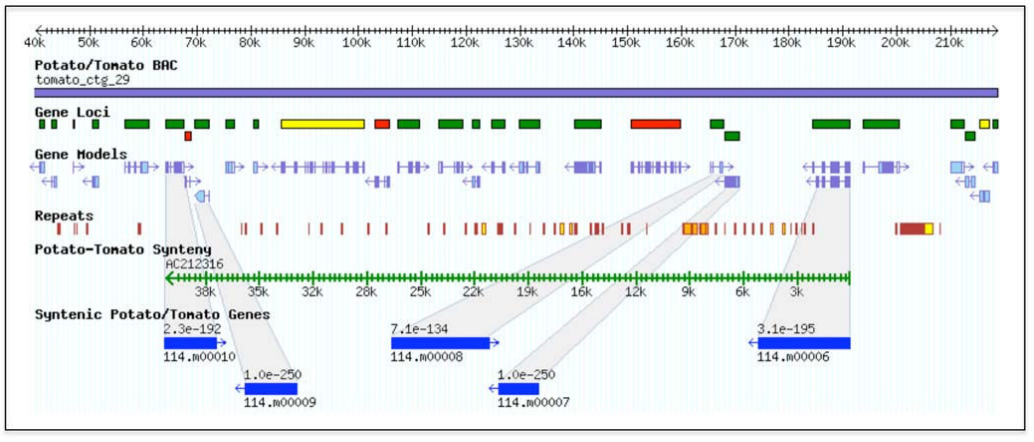
**Insertion/deletions between the potato and the tomato sequences**. Syntenic potato and tomato sequences, along with predicted genes (shown in grey highlights), are shown. Tomato contig 29 is shown at the top of the figure with loci (color coded as described in Figure 1) and gene models. Repetitive sequences in tomato were identified. Potato BAC AC212316 is syntenic with the tomato contig; five potato gene models are conserved in sequence and transcription order with tomato. A large region of non-colinearity (86 kb) is present.

**Figure 5:**
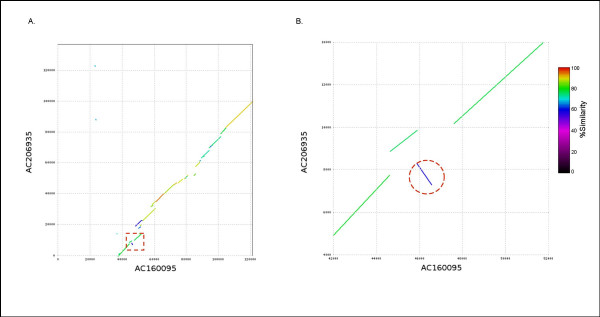
**Micro-inversion within a syntenic region of potato and tomato**. A) Synteny between potato BAC AC206935 and tomato BAC AC160095 is shown with the inverted region marked by a red dotted rectangle B) enlargement of the inverted region with the micro-inversion indicated by the red dotted circle.

In total, we selected 11 potato BACs that were putatively syntenic with tomato; eight candidate syntenic BACs were identified using BES analyses (Set I, see Methods) and three candidate syntenic potato BACs were selected using gene model analyses (Set II, see Methods; Additional Data File 1). The tomato BACs, identified as syntenic to potato BACs, were downloaded either from Genbank or SGN [52] and 14 overlapping tomato BACs were merged into 6 contigs (Additional Data File 1). A total of 1.69 Mb of potato sequence and 2.2 Mb of tomato sequence were used to determine the extent of synteny present between these two *Solanum *species. To assess the sequence similarity at the nucleotide level, we used the program NUCMER with the default settings (i.e., NUCMER command-line options "--minmatch 20 --maxgap 90 --mincluster 65 --breaklen 200") [53], to align these syntenic blocks. Collectively, 515 kb of the tomato and potato sequence could be aligned (Table 4) with high levels of nucleotide identity (89–91%). Although dependent on the portions of the genome the respective BACs represented, up to 73% coverage between syntenic clones was observed. At the gene level, alignment of the potato and tomato protein sequences revealed a high degree of synteny; a total of 98 annotated proteins within the contiguous regions were identified as syntenic. This synteny was sufficient to enable ordering and orientation of contigs within HTG phase 1 potato BACs (Additional Data File 1). However, synteny was not absolute between tomato and potato. In the absence of large insertions/deletions, syntenic regions should have similar length and the large difference in length between some potato and corresponding tomato syntenic regions suggested the existence a bulk insertion or deletion event (Table 4). As shown in Figure 4, an 86 kb insertion/deletion is apparent between potato BAC AC212316 and tomato contig 29. This insertion involves not only repetitive sequences but also non-transposable element-related genes. In addition, micro-scale inversions were observed as shown in Figure 5.

**Table 4:** Statistics on syteny between potato and tomato.

Potato BAC	Tomato Sequence	Match Length (bp)	Average Identity	Synteny Length (bp)	Coverage	Synteny Length Difference (bp)	No. Gene Pairs
AC206935	AC160095	56,255	90%	99,409	57%	-16,036	12
AC151802	AC209589	20,477	91%	43,506	47%	39,432	4
EF514213	AC209589	41,757	90%	151,064	28%	-96,865	11
AC209515	tomato_ctg_27	14,425	90%	112,553	13%	7,387	4
AC212316	tomato_ctg_29	30,979	90%	42,410	73%	85,629	5
AC211135	tomato_ctg_35	72,298	90%	114,572	63%	544	12
AC211296	tomato_ctg_35	71,858	91%	124,673	58%	-24,122	9
AC209518	tomato_ctg_54	49,042	91%	133,407	37%	-31,696	8
AC212966	tomato_ctg_54	83,225	90%	154,451	54%	-7,929	17
AC212553	tomato_ctg_61	11,383	87%	22,653	50%	17,301	7
AC212552	tomato_ctg_98	63,458	89%	131,576	48%	-24,035	9

Each syntenic region consists of a number of matches and gaps The Match Length and Average Identity columns represent the total length and the average sequence identity of the matches in each syntenic block. The length of the syntenic region (i.e., the sum of the lengths of the matches and gaps) and the percentage of the matches over the syntenic blocks in the potato BACs is shown in the columns "Synteny Length" and "Coverage", respectively. The Synteny Length Difference column represents the length difference between the potato and the corresponding tomato syntenic regions. A positive number means that the tomato syntenic region is longer than the corresponding potato syntenic region (i.e., there is an insertion in the tomato BAC or a deletion in the potato BAC) and vice versa. Columns Match Length, Average Identity, Synteny Length, Coverage and Synteny Length Difference are statistics summarized from the sequence alignments using the program NUCMER [53] at the nucleotide level. The No. Gene Pairs column shows the number of gene pairs syntenic identified through the DAGchainer program [68].

### Repetitive content of the potato genome

Using RepeatMasker and the TIGR *Solanum *Repeat Database v3.3, the repeat content of the potato genome as represented by the potato BES (140,259 sequences; 87.14 Mb) dataset and a set of 18 potato BACs (2.20 Mb) was assessed. For comparative purposes, we also assessed the repetitive sequence content in both the set of syntenic tomato BACs (1.69 Mb) and the tomato BES dataset (305,429 sequences; 273.99 Mb). For both potato and tomato, we were able to identify sequences within the BES dataset for five major classes of repetitive sequences: retrotransposons, transposons, telomere-related, ribosomal RNA, and unclassified repetitive sequences suggesting good representation of the genomes were provided by these two datasets. Overall, there were substantially more repeats identified in tomato BES dataset (46.29%) than in potato BES dataset (34.18%; Table 5; Additional Data File 4). In both potato and tomato, more than half of the repeats identified (19.28% and 25.64%, respectively) fell into the unclassified repeat class, most likely due to the lack of characterization of the *Solanum *repeats. Retrotransposon sequences were the most abundant in both potato and tomato BES datasets (11.77% and 14.54%, respectively). However, while there were more Ty3-gypsy type retrotransposons than Ty1-copia retrotransposons (4.60% vs. 1.82%) in the potato BES dataset, the margin between these two types in tomato BES dataset was much smaller (Ty3-gypsy 4.61% vs. Ty1-copia 3.75%). Examination of individual libraries within the potato and tomato BES datasets (Additional Data File 4) indicated that this was not due to an over-representation of Ty1-copia elements in tomato in the *Mbo*I library but rather due to a decreased representation of Ty1-copia elements in both the potato *Hin*dIII and *Eco*RI datasets compared to tomato. Overall, the percent of transposon sequences found in potato versus tomato BES dataset was comparable (1.32% vs. 1.39%). This is due to the similar representation of unclassified transposons in both tomato and potato. Representation of Ac/Ds transposons was skewed between potato and tomato (0.13% vs 0.05%), which was attributable to a much higher representation of Ac/Ds elements in the potato *Hin*dIII library compared to the tomato *Hin*dIII library (Additional Data File 4).

**Table 5:** Classification of repetitive sequences in the potato and tomato genome.

Classification	Potato BAC			Tomato BAC	Potato BES	Tomato BES
				
	Total	Syntenic	Non-syntenic			
Transposable elements	12.27%	8.51%	13.19%	10.66%	13.09%	15.93%
Retrotransposons	9.58%	7.22%	11.19%	8.32%	11.77%	14.54%
Ty1-copia	1.92%	1.15%	1.79%	3.48%	1.82%	3.75%
Ty3-gypsy	3.34%	4.09%	6.33%	0.99%	4.60%	4.61%
LINE	1.09%	0.46%	0.72%	0.77%	0.80%	0.44%
SINE	0.54%	0.30%	0.46%	0.47%	0.27%	0.12%
Unclassified Retrotransposons	2.70%	1.22%	1.90%	2.61%	4.29%	5.64%
						
Transposons	2.69%	1.29%	1.99%	2.35%	1.32%	1.39%
Ac/Ds	0.13%	0.13%	0.21%	0.01%	0.13%	0.05%
CACTA, En/Spm	0.03%	0.05%	0.07%	0.00%	0.03%	0.01%
Unclassified Transposons	2.53%	1.11%	1.71%	2.34%	1.15%	1.32%
						
Telomere-related	0.04%	0.00%	0.01%	0.00%	1.31%	0.73%
Telomere-associated	0.04%	0.00%	0.01%	0.00%	0.49%	0.72%
Telomere	0.00%	0.00%	0.00%	0.00%	0.82%	0.01%
						
Ribosomal RNA genes	0.07%	0.08%	0.12%	0.02%	0.50%	3.99%
45S rDNA	0.02%	0.03%	0.05%	0.01%	0.43%	3.96%
5S rDNA	0.05%	0.05%	0.07%	0.02%	0.07%	0.03%
						
Unclassified	13.51%	12.38%	19.18%	11.61%	19.28%	25.64%
						
Total	25.90%	20.97%	32.50%	22.30%	34.18%	46.29%

**Figure 6:**
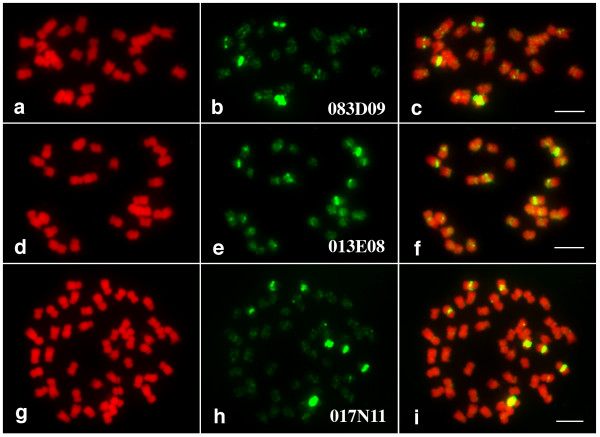
**FISH analysis of BACs that contain telomeric repeat sequences **RHPOTKEY083D09 and RHPOTKEY013E08, which contain telomeric repeat at one end, and RHPOTKEY017N11, which contains telomeric repeats at both ends. (a, d, g) Chromosomes prepared from USW1 (a, d) and Katahdin (g), respectively. (b, e, h) FISH signals derived from the BAC clones. (c, f, i) Images merged from chromosomes and FISH signals. Bars = 5 μm.

Surprisingly, there were nearly twice as many telomere-related repeat sequences identified in the potato BES dataset compared to that of the tomato BES (1.31% in potato vs 0.73% in tomato) with the major difference occurring in the telomere repeat representation (0.82% in potato, 0.01% in tomato, Table 5). While telomeric sequences are enriched in the telomeres, they can be found in centromeric and pericentromeric regions [38] and clearly, based on their abundance in the potato BES dataset, are prevalent in the potato genome. We selected two potato BAC clones which contain telomeric repeats on both end sequences and six BAC clones in which only one of the ends contained the telomeric-repetitive sequences (Additional Data File 5). These BACs were used in fluorescent *in situ *hybridization (FISH) studies to assess where on the chromosomes these repetitive sequences localized. As shown in Figure 6, these clones did not generate unambiguous signals at the telomeres of potato chromosomes but produced major signals in the centromeric and pericentromeric regions of several potato chromosomes. These results showed that these potato BACs were most likely derived from centromeric rather than telomeric regions of potato chromosomes.

A significant amount of rDNA sequences (3.99%) were detected in the tomato BES dataset while rDNA sequences found in potato BAC ends (0.50%) were minimal in comparison. The tomato BES were derived from three libraries constructed with *Eco*RI, *Hin*dIII and *Mbo*I restriction enzymes while the potato BES were derived from two libraries constructed with *Eco*RI and *Hin*dIII. Multiple *Eco*RI and *Mbo*I restriction sites are present in both the tomato and potato rDNA sequence (data not shown) and for the potato BES dataset, the ratio between *Eco*RI BES and *Hin*dIII BES is 0.70 (57,778/82,481). Therefore, there should be ample detection of rDNA sequences in the potato BES datasets suggesting that there may be a bias in overall rDNA content between potato and tomato. Analysis of individual libraries for potato and tomato confirmed this finding (Additional Data File 4). The rDNA sequences in potato are reported to be on chromosomes 1 (5S; [54]) and 2 (45S; nucleolar organizing region, [54,55]). It has been reported that rDNA content differs between potato and tomato with tomato having more rDNA than potato [36,56]. Thus, it is likely that the sampling of rDNA sequences, as reflected by BES survey sequencing, is reflective of a true rDNA content difference in the nuclear genomes of tomato and potato.

To contrast with the short BES-derived genome sequence, a total of 18 phase 2 and 3 potato BACs (2.20 Mb) and 16 tomato BACs (1.69 Mb, in 8 contigs/BACs) were analyzed for repetitive sequence content. Overall, the repetitive sequence fractions identified were comparable between potato and tomato BACs (25.90% vs. 22.30%). Similar to that observed with the BES datasets, more than half of the repeats identified in both the potato and tomato BAC sequences were unclassified (13.51% vs. 11.61%, respectively) while retrotransposon sequences were the most abundant characterized repetitive element in both potato and tomato BACs (9.58% vs. 8.32%, respectively). As observed with the potato BES dataset, there were more Ty3-gypsy type retrotransposons than Ty1-copia retrotransposons (3.34% vs. 1.92%) in the potato BACs. However, in contrast to that observed in the tomato BES dataset, more Ty1-copia than Ty3-gypsy type retrotransposons were present in tomato BAC sequences (0.99% Ty3-gypsy vs. 3.48% Ty1-copia). Interestingly, more transposon sequences were found in potato and tomato BAC sequences (2.69% vs 2.35%, respectively) than in the BES datasets (1.32% potato vs 1.39% tomato BES). Not surprisingly, there were almost no telomeric-related repetitive sequences or rDNA sequences identified in either potato or tomato BAC sequences. The lack of these sequences in the limited BACs examined is reflective of the euchromatic nature of the tomato BACs and their syntenic potato counterparts.

For potato, the overall percentages of repetitive sequences identified in the BAC and BES datasets were relatively comparable (total repeats: BES 34.18% vs. BAC 25.90%) and is consistent with the fact that nearly half of the potato BACs sequenced in this study (7/19 BACs) were randomly selected and reflect characteristics of the entire potato BAC library and genome. However, for tomato, the overall composition of repetitive sequences in the BES and BAC datasets differed significantly (total repeats: BES 46.29% vs. BAC 22.30%). This is attributable to the fact that the BACs sequenced by the Tomato Genome Initiative [52] are preferentially selected from the euchromatic regions which contain less repetitive sequences than the heterochromatin regions of the genome [48].

Certainly, identification of differences in relative composition of repetitive sequences between potato and tomato is not novel, however, the large difference in total repetitive sequence content between tomato and potato is surprising considering that these two *Solanum *species diverged less than 12 Million Years Ago [57]. The haploid genome size of tomato and potato differ with tomato reported to be 950 Mb while that of potato is 865 Mb (range 798–931 Mb; [27]). Thus, with 34.2% and 46.3% repetitive sequences in potato and tomato, respectively, the total repetitive sequence space within the whole genome would be 296 Mb (potato) and 440 Mb (tomato) leaving a comparable non-repetitive fraction of their genomes of 569 Mb in potato and 510 Mb in tomato. This higher level of repetitive sequence is consistent with our finding of a higher frequency of matches within the potato BES to a Solanaceae EST compared to the tomato BES (5.5% vs 3.8%, respectively). Thus, the repetitive sequences within their respective genomes not only diverged in terms of classes of sequences but also in number leading to a biased amplification of repetitive sequences in tomato compared to potato.

## Conclusion

We report on a large set of genomic sequences representing 10.2% of the potato genome. Using comparative analyses with solanaceous species we were able to demonstrate the utility and power of comparative genomics to not only annotate potato genomic sequences but also to assist in genome sequencing efforts among the Solanaceae. While we were able to confirm synteny on a genome scale with segments of the tomato and potato genome > 100 kb, we have also demonstrated that synteny is not absolute and that insertions/deletions as well as micro-inversions have occurred since the divergence of potato and tomato. More strikingly, the repetitive sequence content and composition of potato and tomato have diverged with impacts seen on genome architecture at both the macro- and the micro-level as evidenced through differences in telomeric-repetitive sequences and rDNA content and in interruption of synteny through transposition of retrotransposons. Our data are consistent with previous reports on repetitive sequences [36-42] which show divergence of this fraction of the genome within the Solanaceae. These data clearly suggest that while these two solanaceous genomes can be cross-leveraged for analysis of gene content and order, they are not interchangeable with respect to all genomic features.

## Materials

### Sequences used in this study

Potato genome sequences (BES and BAC sequences) generated in this study are described below. Tomato BAC end sequences (305,429 sequences, 273.99 Mb total) were downloaded from the GSS division of Genbank on Nov. 12, 2007. EST collections for the solanaceous species were obtained from the TIGR Plant Transcript Assemblies project ([49,58]; dated on 11/20/2007). The release versions used in this study are shown in Additional Data File 3. The tomato BAC sequences were downloaded from Genbank and SGN [52] on Oct. 29, 2007, and were merged into a set of 518 unique tomato BACs.

### Sequencing methods

The RHPOTKEY BAC library was constructed from RH parent *Solanum tuberosum *var. RH89-039-16 using *Hin*dIII and *Eco*RI restriction enzymes (C. Bachem, Pers. Comm., [28]). Templates were prepared using a high throughput alkaline lysis method, sequenced on ABI 3730 × l sequencers using TF and TR primers using standard high throughput sequencing methods, and processed with Paracel Trace Tuner [59]. All sequences were trimmed to remove vector, low-quality, and *E. coli *sequences using Lucy [60] and iterative runs of the TIGR Seqclean Tool [61]. All potato BAC end sequences have been submitted to the GSS division of Genbank with accession numbers EI367122-EI91525, EI812397-EI846477, and ER788642-ER870415.

Potato BAC DNA was isolated using the Sigma Phase Prep BAC DNA kit (Sigma, St Louis, MO) according to manufacturer's protocol. Approximately 7.5 ug was used for library construction. Samples were treated overnight with 100 U of Plasmid-Safe ATP-Dependent Dnase (Epicenter, Madison, WI) to remove contaminating bacterial chromosomal DNA and nebulized. Sheared DNA was precipitated and polished using the DNATerminator End Repair Kit following the manufacturer's protocol (Lucigen, Middleton, WI). Samples were electrophoresed on a 1.0% low melting point agarose and fragments in the range of 3–6 kb were selected for ligation into the pSMART-HCKan vector (Lucigen, Middleton, WI). Templates from the shotgun libraries were sequenced using TX and TY primers as described by Lucigen using standard high throughput sequencing methods on ABI 3730 × l sequencers. Sequences were trimmed as described above for the BAC end sequences and assembled with Celera Assembler [62]. Potato BAC sequences have been deposited in the HTG division of Genbank under accession numbers AC204499, AC204500, AC206931-AC206936, AC209514-AC209520, AC212037, AC212316, AC212552, and AC212966.

### Fluorescent *in situ *hybridization

Potato variety Katahdin (2n = 48) and a haploid clone USW1 (2n = 24) derived from Katahdin were used in FISH analysis. The FISH procedure followed published protocols [63]. Briefly, BAC DNA was isolated and labeled with Biotin-UTP. Hybridization signals were detected FITC-conjugated avidin. Chromosomes were counterstained by 4', 6-diamidino-2phenylindole (DAPI) and were pseudocolored in red. Images were captured digitally using a SenSys CCD (charge coupled device) camera attached to an Olympus BX60 epifluorescence microscope. The CCD camera was controlled using IPLab Spectrum v3.1 software (Signal Analytics, Vienna, VA) on a Macintosh computer.

### Annotation

The potato BACs and the syntenic tomato contigs were annotated in parallel. First, the potato and tomato BACs were masked for repetitive sequences using RepeatMasker with a modified TIGR *Solanum *Repeat Database v3.3 in which miniature inverted repeat transposable elements (MITEs) and non-transposable element-related repeats were excluded. Second, gene models were predicted using the *ab initio *gene finder FGENESH (dicot matrix; [64]) and were updated using transcript evidence (ESTs, cDNAs) with the Program to Assemble Spliced Alignments [65]. Moreover, the gene structures were manually inspected and some aberrant models, e.g., overlapping/nested or short (< 50 amino acids) genes, were removed. Third, gene function was assigned based on sequence identity to proteins within an in-house non-redundant protein database and/or the presence of Pfam domain(s), in a similar manner as reported previously for annotation of the rice genome [66]. Gene functions were classified into three categories: "known/putative", "expressed" or "hypothetical". Genes in which functional assignments could be assigned based on sequence similarity to a known protein or the presence of a Pfam domain above the trusted cutoff score (unique for each Pfam domain) were annotated as encoding either a known or putative protein; the remaining gene models for which no sequence similarity or Pfam domain evidence was available were annotated as encoding an "expressed protein" if cognate transcript support was available or "hypothetical protein" if cognate transcript support was absent.

The solanaceous transcript assemblies (downloaded from [58]) were searched against the potato BACs using the program GAP2 [67]. High quality alignments were defined as having sequence identity ≥ 80% and coverage ≥ 70% of the length of the Transcript Assembly. Only alignments meeting these cutoff criteria were used in downstream analyses and a solanaceous transcript was considered to support the *ab initio*-based annotation if the spliced alignment of the transcript overlapped a minimum of 100 bp with the gene model.

### Identification of candidate syntenic tomato sequences

We utilized two methods to identify potential syntenic tomato-potato sequences. For Set I, tomato BACs were downloaded either from Genbank or SGN [52] and 14 overlapping tomato BACs were merged into 6 contigs to facilitate alignment and mapping to the potato BES. The potato BES were repeat masked and mapped to the tomato contigs using the program BLASTN with an E value cutoff of ≤ 1e-5. Paired potato BES were selected if they mapped to the same tomato contig in the correct orientation and within an expected intervening distance (50~200 kb). In total, 52 potato BACs were identified as candidate syntenic clones; eight potato BAC clones were sequenced. It is possible that BACs either from chromosome 6 or other chromosomes in the potato genome are syntenic with tomato BACs available in the public domain. To address this issue, we utilized the *ab initio *gene finder, FGENESH [64] to predict genes in the 18 phase 2 and 3 potato BACs and the 518 tomato BACs and searched these gene models against each other using BLASTP. The DAGchainer program [68] was employed to identify syntenic gene blocks between the potato and tomato contigs; putative syntenic potato-tomato BACs identified with this approach were termed Set II.

Synteny between tomato and potato was examined at the nucleotide and the protein level. Genomic comparisons at the nucleotide level utilized the NUCMER program [53]. Syntenic gene blocks between potato and tomato contigs were generated by the BLASTP/DAGchainer [68] pipeline using the predicted protein sequences from the semi-automated annotation pipeline with improved gene structures/models rather than the *ab initio *FGENESH predictions.

### Repeat database construction

Publicly available sequences were searched to expand our existing TIGR *Solanum *Repeat Database [69,70]. New Solanaceae repetitive sequences were first collected from Genbank and used to update the TIGR Solanaceae Repeat Database. The TIGR Solanaceae Repeat Database was then searched against *Solanum *BAC sequences (41 non-tomato *Solanum *BACs and 301 tomato BACs, 40.05 Mb total sequence) from GenBank and the SGN [52] using RepeatMasker ([71] with a cut-off score of 225 which should not yield false positives). Sequences within the BACs that matched a repetitive sequence in the TIGR Solanaceae Repeat Database with ≥ 75% identity and ≥ 95% overall length were excised, coded [72], and combined with other *Solanum *repetitive sequences in the TIGR Solanaceae Repeat Database. Lastly, the same set of *Solanum *BAC sequences was searched with the *de novo *repetitive sequence finding algorithm, RepeatScout [73]. Low-complexity sequences in the RepeatScout-generated fasta-formated sequence output were filtered out. To prevent inclusion of paralogous protein coding genes, all RepeatScout-generated sequences with similarity to known proteins or Pfam domains were identified and removed. All remaining repetitive sequences were coded based on the similarity with known repetitive sequences and added to the *Solanum *repetitive sequences to create the TIGR *Solanum *Repeat Database v3.3.

### Repetitive sequence identification

Potato and tomato BAC end sequences (BES, 87.14 Mb and 273.99 Mb, respectively) and BAC sequences used in this study (2.20 Mb and 1.69 Mb, respectively) were searched against the TIGR *Solanum *Repeat Database v3.3 using RepeatMasker with a cut-off score of 225. Genomic sequences were quantified based RepeatMasker matches to the TIGR Solanum Repeat Database v3.3 sequences and quantitated at the sub-class level [70].

## Abbreviations

Bacterial artificial chromosome: BAC; BAC end sequence: BES; Fluorescent *in situ *hybridization: FISH; Long Terminal Repeat: LTR; Transcript Assembly: TA.

## Authors' contributions

WZ and SO contributed equally to this work. WZ, SO, and HV conducted analyses of the sequence and annotation and participated in writing the manuscript. KO'B sequenced the BAC clones. MI and JJ conducted the FISH experiments and participated in writing the manuscript. CRB designed the experiments, oversaw the execution of the experiments and analyses, and participated in writing the manuscript.

## Supplementary Material

Additional file 1 Potato BACs used in this study. Click here for file

Additional file 2 Construction of contigs from tomato BACs. Click here for file

Additional file 3 Solanaceae Transcript Assemblies used in this study. Click here for file

Additional file 4 Repetitive sequence content in different BAC libraries Click here for file

Additional file 5 Sequence of end sequences from potato BAC clones used in fluorescent in situ hybridizations. The telomeric repetitive sequences are highlighted. Click here for file

## References

[B1] Bradshaw JE, Hackett CA, Pande B, Waugh R, Bryan GJ (2008). QTL mapping of yield, agronomic and quality traits in tetraploid potato (*Solanum tuberosum *subsp. *tuberosum*). Theor Appl Genet.

[B2] Kloosterman B, Vorst O, Hall RD, Visser RG, Bachem CW (2005). Tuber on a chip: differential gene expression during potato tuber development. Plant Biotechnol J.

[B3] Hofvander P, Andersson M, Larsson CT, Larsson H (2004). Field performance and starch characteristics of high-amylose potatoes obtained by antisense gene targeting of two branching enzymes. Plant Biotechnol J.

[B4] Li XQ, De Jong H, De Jong DM, De Jong WS (2005). Inheritance and genetic mapping of tuber eye depth in cultivated diploid potatoes. Theor Appl Genet.

[B5] Ducreux LJ, Morris WL, Hedley PE, Shepherd T, Davies HV, Millam S, Taylor MA (2005). Metabolic engineering of high carotenoid potato tubers containing enhanced levels of beta-carotene and lutein. J Exp Bot.

[B6] Romer S, Lubeck J, Kauder F, Steiger S, Adomat C, Sandmann G (2002). Genetic engineering of a zeaxanthin-rich potato by antisense inactivation and co-suppression of carotenoid epoxidation. Metab Eng.

[B7] Huang S, Vossen EA van der, Kuang H, Vleeshouwers VG, Zhang N, Borm TJ, van Eck HJ, Baker B, Jacobsen E, Visser RG (2005). Comparative genomics enabled the isolation of the R3a late blight resistance gene in potato. Plant J.

[B8] Kuang H, Wei F, Marano MR, Wirtz U, Wang X, Liu J, Shum WP, Zaborsky J, Tallon LJ, Rensink W, Lobst S, Zhang P, Tornqvist CE, Tek A, Bamberg J, Helgeson J, Fry W, You F, Luo MC, Jiang J, Robin Buell C, Baker B (2005). The R1 resistance gene cluster contains three groups of independently evolving, type I R1 homologues and shows substantial structural variation among haplotypes of *Solanum demissum*. Plant J.

[B9] Rauscher GM, Smart CD, Simko I, Bonierbale M, Mayton H, Greenland A, Fry WE (2006). Characterization and mapping of RPi-ber, a novel potato late blight resistance gene from *Solanum berthaultii*. Theor Appl Genet.

[B10] Song J, Bradeen JM, Naess SK, Raasch JA, Wielgus SM, Haberlach GT, Liu J, Kuang H, Austin-Phillips S, Buell CR, Helgeson JP, Jiang J (2003). Gene RB cloned from *Solanum bulbocastanum *confers broad spectrum resistance to potato late blight. Proc Natl Acad Sci USA.

[B11] Ballvora A, Jocker A, Viehover P, Ishihara H, Paal J, Meksem K, Bruggmann R, Schoof H, Weisshaar B, Gebhardt C (2007). Comparative sequence analysis of *Solanum *and Arabidopsis in a hot spot for pathogen resistance on potato chromosome V reveals a patchwork of conserved and rapidly evolving genome segments. BMC Genomics.

[B12] Ronning CM, Stegalkina SS, Ascenzi RA, Bougri O, Hart AL, Utterbach TR, Vanaken SE, Riedmuller SB, White JA, Cho J, Pertea GM, Lee Y, Karamycheva S, Sultana R, Tsai J, Quackenbush J, Griffiths HM, Restrepo S, Smart CD, Fry WE, Hoeven R Van Der, Tanksley S, Zhang P, Jin H, Yamamoto ML, Baker BJ, Buell CR (2003). Comparative analyses of potato expressed sequence tag libraries. Plant Physiol.

[B13] Flinn B, Rothwell C, Griffiths R, Lague M, DeKoeyer D, Sardana R, Audy P, Goyer C, Li XQ, Wang-Pruski G, Regan S (2005). Potato expressed sequence tag generation and analysis using standard and unique cDNA libraries. Plant Mol Biol.

[B14] Rensink W, Hart A, Liu J, Ouyang S, Zismann V, Buell CR (2005). Analyzing the potato abiotic stress transcriptome using expressed sequence tags. Genome.

[B15] Song J, Dong F, Jiang J (2000). Construction of a bacterial artificial chromosome (BAC) library for potato molecular cytogenetics research. Genome.

[B16] Chen Q, Sun S, Ye Q, McCuine S, Huff E, Zhang HB (2004). Construction of two BAC libraries from the wild Mexican diploid potato, *Solanum pinnatisectum*, and the identification of clones near the late blight and Colorado potato beetle resistance loci. Theor Appl Genet.

[B17] Rensink WA, Iobst S, Hart A, Stegalkina S, Liu J, Buell CR (2005). Gene expression profiling of potato responses to cold, heat, and salt stress. Funct Integr Genomics.

[B18] van Os H, Andrzejewski S, Bakker E, Barrena I, Bryan GJ, Caromel B, Ghareeb B, Isidore E, de Jong W, van Koert P, Lefebvre V, Milbourne D, Ritter E, Voort JN van der, Rousselle-Bourgeois F, van Vliet J, Waugh R, Visser RG, Bakker J, van Eck HJ (2006). Construction of a 10,000-marker ultradense genetic recombination map of potato: providing a framework for accelerated gene isolation and a genomewide physical map. Genetics.

[B19] Campbell M, Segear E, Beers L, Knauber D, Suttle J (2008). Dormancy in potato tuber meristems: chemically induced cessation in dormancy matches the natural process based on transcript profiles. Funct Integr Genomics.

[B20] Feingold S, Lloyd J, Norero N, Bonierbale M, Lorenzen J (2005). Mapping and characterization of new EST-derived microsatellites for potato (*Solanum tuberosum *L.). Theor Appl Genet.

[B21] Nielsen KL, Gronkjaer K, Welinder KG, Emmersen J (2005). Global transcript profiling of potato tuber using LongSAGE. Plant Biotechnol J.

[B22] Schafleitner R, Gutierrez Rosales RO, Gaudin A, Alvarado Aliaga CA, Martinez GN, Tincopa Marca LR, Bolivar LA, Delgado FM, Simon R, Bonierbale M (2007). Capturing candidate drought tolerance traits in two native Andean potato clones by transcription profiling of field grown plants under water stress. Plant Physiol Biochem.

[B23] Stupar RM, Bhaskar PB, Yandell BS, Rensink WA, Hart AL, Ouyang S, Veilleux RE, Busse JS, Erhardt RJ, Buell CR, Jiang J (2007). Phenotypic and transcriptomic changes associated with potato autopolyploidization. Genetics.

[B24] Van Damme EJ, Barre A, Rouge P, Peumans WJ (2004). Potato lectin: an updated model of a unique chimeric plant protein. Plant J.

[B25] Watkinson JI, Hendricks L, Sioson AA, Heath LS, Bohnert HJ, Grene R (2008). Tuber development phenotypes in adapted and acclimated, drought-stressed *Solanum tuberosum *ssp. *andigena *have distinct expression profiles of genes associated with carbon metabolism. Plant Physiol Biochem.

[B26] Rensink WA, Lee Y, Liu J, Iobst S, Ouyang S, Buell CR (2005). Comparative analyses of six solanaceous transcriptomes reveal a high degree of sequence conservation and species-specific transcripts. BMC Genomics.

[B27] Arumuganathan K, Earle E (1991). Nuclear DNA content of some important plant species. Plant Molecular Biology Reporter.

[B28] The Potato Genome Sequencing Consortium http://potatogenome.net.

[B29] United States Department of Agriculture National Agricultural Statistics Service http://usda.mannlib.cornell.edu/usda/nass/CropValuSu//2000s/2007/CropValuSu-02-15-2007.txt.

[B30] Zamir D, Tanksley S (1988). Tomato genome is comprised of largely of fast-evolving, low copy-number sequences. Molecular General Genetics.

[B31] Bonierbale MW, Plaisted RL, Tanksley SD (1988). RFLP maps based on a common set of clones reveal modes of chromosomal evolution in potato and tomato. Genetics.

[B32] De Jong WS, Eannetta NT, De Jong DM, Bodis M (2004). Candidate gene analysis of anthocyanin pigmentation loci in the Solanaceae. Theor Appl Genet.

[B33] Livingstone KD, Lackney VK, Blauth JR, van Wijk R, Jahn MK (1999). Genome mapping in *Capsicum *and the evolution of genome structure in the solanaceae. Genetics.

[B34] Tanksley SD, Ganal MW, Prince JP, de Vicente MC, Bonierbale MW, Broun P, Fulton TM, Giovannoni JJ, Grandillo S, Martin GB, Messeguer R, Miller JC, Miller L, Paterson AH, Pineda O, Roder MS, Wing RA, Wu W, Young ND (1992). High density molecular linkage maps of the tomato and potato genomes. Genetics.

[B35] Thorup TA, Tanyolac B, Livingstone KD, Popovsky S, Paran I, Jahn M (2000). Candidate gene analysis of organ pigmentation loci in the Solanaceae. Proc Natl Acad Sci USA.

[B36] Schweizer G, Borisjuk N, Borisjuk L, Stafler M, Stelzer T, Schilde L, Hemleben V (1993). Molecular analysis of highly repeated genome fractions in *Solanum *and their use as markers for the characterization of species and cultivars. Theor Appl Genet.

[B37] Ganal MW, Lapitan N, Tanksley S (1988). A molecular and cytogenetic survey of major repeated DNA sequences in tomato (*Lycopersicon esculentum*). Mol Gen Genet.

[B38] Tek AL, Jiang J (2004). The centromeric regions of potato chromosomes contain megabase-sized tandem arrays of telomere-similar sequence. Chromosoma.

[B39] Tek AL, Song J, Macas J, Jiang J (2005). Sobo, a recently amplified satellite repeat of potato, and its implications for the origin of tandemly repeated sequences. Genetics.

[B40] Stupar RM, Song J, Tek AL, Cheng Z, Dong F, Jiang J (2002). Highly condensed potato pericentromeric heterochromatin contains rDNA-related tandem repeats. Genetics.

[B41] Schweizer G, Ganal MW, Ninnemann H, Hemleben V (1988). Species-specific DNA sequences for identification of somatic hybrids between *Lycopersicon esculentum *and *Solanum acaule*. Theor Appl Genet.

[B42] Gebhardt C, Eberle B, Leonards-Schippers C, Walkemeier B, Salamini F (1995). Isolation, characterization and RFLP linkage mapping of a DNA repeat family of *Solanum spegazzinii *by which chromosome ends can be localized on the genetic map of potato. Genet Res.

[B43] Arabidopsis Genome Initiative (2000). Analysis of the genome sequence of the flowering plant *Arabidopsis thaliana*. Nature.

[B44] Tuskan GA, Difazio S, Jansson S, Bohlmann J, Grigoriev I, Hellsten U, Putnam N, Ralph S, Rombauts S, Salamov A, Schein J, Sterck L, Aerts A, Bhalerao RR, Bhalerao RP, Blaudez D, Boerjan W, Brun A, Brunner A, Busov V, Campbell M, Carlson J, Chalot M, Chapman J, Chen GL, Cooper D, Coutinho PM, Couturier J, Covert S, Cronk Q (2006). The genome of black cottonwood, *Populus trichocarpa *(Torr. & Gray). Science.

[B45] Jaillon O, Aury JM, Noel B, Policriti A, Clepet C, Casagrande A, Choisne N, Aubourg S, Vitulo N, Jubin C, Vezzi A, Legeai F, Hugueney P, Dasilva C, Horner D, Mica E, Jublot D, Poulain J, Bruyere C, Billault A, Segurens B, Gouyvenoux M, Ugarte E, Cattonaro F, Anthouard V, Vico V, Del Fabbro C, Alaux M, Di Gaspero G, Dumas V (2007). The grapevine genome sequence suggests ancestral hexaploidization in major angiosperm phyla. Nature.

[B46] International Rice Genome Sequencing Project (2005). The map-based sequence of the rice genome. Nature.

[B47] Wu TD, Watanabe CK (2005). GMAP: a genomic mapping and alignment program for mRNA and EST sequences. Bioinformatics.

[B48] Mueller LA, Tanksley SD, Giovannoni JJ, van Eck J, Stack S, Choi D, Kim BD, Chen M, Cheng Z, Li C, Ling H, Xue Y, Seymour G, Bishop G, Bryan G, Sharma R, Khurana J, Tyagi A, Chattopadhyay D, Singh NK, Stiekema W, Lindhout P, Jesse T, Lankhorst RK, Bouzayen M, Shibata D, Tabata S, Granell A, Botella MA, Giuliano G, Frusciante L, Causse M, Zamir D (2005). The Tomato Sequencing Project, the first cornerstone of the International Solanaceae Project (SOL). Comp Func Genomics.

[B49] Childs KL, Hamilton JP, Zhu W, Ly E, Cheung F, Wu H, Rabinowicz PD, Town CD, Buell CR, Chan AP (2007). The TIGR Plant Transcript Assemblies database. Nucleic Acids Res.

[B50] Gebhardt C, Ritter E, Barone A, Debener T, Walkemeier B, Schachtschabel U, Kaufmann H, Thompson RD, Bonierbale MW, Ganal MW, Tanksley SD, Salamini F (1991). RFLP maps of potato an their alignment with the homoeologous tomato genome. Theor Appl Genet.

[B51] Doganlar S, Frary A, Daunay MC, Lester RN, Tanksley SD (2002). A comparative genetic linkage map of eggplant (*Solanum melongena*) and its implications for genome evolution in the solanaceae. Genetics.

[B52] Solanaceae Genome Network http://soldb.cit.cornell.edu/.

[B53] Kurtz S, Phillippy A, Delcher AL, Smoot M, Shumway M, Antonescu C, Salzberg SL (2004). Versatile and open software for comparing large genomes. Genome Biol.

[B54] Dong F, Song J, Naess SK, Helgeson JP, Gebhardt C, Jiang J (2000). Development and applications of a set of chromosome-specific cytogenetic DNA markers in potato. Theor Appl Genet.

[B55] Yeh BP, Peloquin SJ (1965). Pachytene chromosomes of the potato (*Solanum tuberosum*, Group Andigena). Amer Jour Bot.

[B56] Komarova NY, Grabe T, Huigen DJ, Hemleben V, Volkov RA (2004). Organization, differential expression and methylation of rDNA in artificial *Solanum *allopolyploids. Plant Mol Biol.

[B57] Moniz de Sa M, Drouin G (1996). Phylogeny and substitution rates of angiosperm actin genes. Mol Biol Evol.

[B58] TIGR Plant Transcript Assemblies Database http://plantta.tigr.org.

[B59] Trace Tuner. http://sourceforge.net/projects/tracetuner.

[B60] Chou HH, Holmes MH (2001). DNA sequence quality trimming and vector removal. Bioinformatics.

[B61] TIGR Seqclean Tool http://compbio.dfci.harvard.edu/tgi/.

[B62] Venter JC, Adams MD, Myers EW, Li PW, Mural RJ, Sutton GG, Smith HO, Yandell M, Evans CA, Holt RA, Gocayne JD, Amanatides P, Ballew RM, Huson DH, Wortman JR, Zhang Q, Kodira CD, Zheng XH, Chen L, Skupski M, Subramanian G, Thomas PD, Zhang J, Gabor Miklos GL, Nelson C, Broder S, Clark AG, Nadeau J, McKusick VA, Zinder N (2001). The sequence of the human genome. Science.

[B63] Jiang J, Hulbert SH, Gill BS, Ward DC (1996). Interphase fluorescence in situ hybridization mapping: a physical mapping strategy for plant species with large complex genomes. Mol Gen Genet.

[B64] Salamov AA, Solovyev VV (2000). Ab initio gene finding in Drosophila genomic DNA. Genome Res.

[B65] Haas BJ, Delcher AL, Mount SM, Wortman JR, Smith RK, Hannick LI, Maiti R, Ronning CM, Rusch DB, Town CD, Salzberg SL, White O (2003). Improving the Arabidopsis genome annotation using maximal transcript alignment assemblies. Nucleic Acids Res.

[B66] Ouyang S, Zhu W, Hamilton J, Lin H, Campbell M, Childs K, Thibaud-Nissen F, Malek RL, Lee Y, Zheng L, Orvis J, Haas B, Wortman J, Buell CR (2007). The TIGR Rice Genome Annotation Resource: improvements and new features. Nucleic Acids Res.

[B67] Huang X, Adams MD, Zhou H, Kerlavage AR (1997). A tool for analyzing and annotating genomic sequences. Genomics.

[B68] Haas BJ, Delcher AL, Wortman JR, Salzberg SL (2004). DAGchainer: a tool for mining segmental genome duplications and synteny. Bioinformatics.

[B69] TIGR Plant Repeat Database http://www.tigr.org/tdb/e2k1/plant.repeats.

[B70] Ouyang S, Buell CR (2004). The TIGR Plant Repeat Databases: a collective resource for the identification of repetitive sequences in plants. Nucleic Acids Res.

[B71] RepeatMasker. http://www.repeatmasker.org/.

[B72] TIGR Plant Repeat Database http://www.tigr.org/tdb/e2k1/plant.repeats/repeat.code.shtml.

[B73] Price AL, Jones NC, Pevzner PA (2005). De novo identification of repeat families in large genomes. Bioinformatics.

