# Supplementary material for: Analysis of 90 Mb of the potato genome reveals conservation of gene structures and order with tomato but divergence in repetitive sequence composition
Source: BMC Genomics. 2008 Jun 13;9:286. doi: 10.1186/1471-2164-9-286 (PMC2442093; doi:10.1186/1471-2164-9-286)
Supplement: Additional file 5 — Sequence of end sequences from potato BAC clones used in fluorescent in situ hybridizations. The telomeric repetitive sequences are highlighted. [file 1471-2164-9-286-S5.doc]

Additional Data File 5. Sequence of end sequences from potato BAC clones used in fluorescent in situ hybridizations.

**>POTJ617TF forward BES of** 083D09

**AGGGTTTTATGTTTAGGGTTTTATCTAAGTATTTAGTGTTTGGGGCTTTGGATTTTTTAG**

**GGTTAGGATTCTTAGGGTTTAATCGGTTTAGGGTTTCGGTGTTAAGTTTTTTTTGTGTTT**

**TTAGGGTTTAACATATACGATTTAGGACTTAATAGGGTTTAGAGCTTAGATTTTTTCTAA**

**CTTAAAGGATTTTGGGGTTTTTTAAGGTTAACTATTTCTGAGGGTTTTTAGGGTTTTGGA**

**TTTACACTTAGGGTTTTTTGTGTTGTAAGTTTAGGGTTTATAGATATTTTGGATTTAGGG**

**TTTAGATCTTATGTTTTGTAGGGGTTTTAAATGATTTTTCGGGGTTTTATAGGGTTAGTA**

**GGATTTAGGGTTTCTTAAGGTTTAAGGTTTAGTGTTTCTTACGGGTTTGAGTTTAGGATT**

**TGGGTTTTGGATTCAGGGTTGATGGATTAGTGTTTTTTTAGATTTAAGGATTTAGGTTAA**

**GTTTTTATCTAGGTTTTTAGGGCTTACGGATTTGGGTTTTTTATGTTAAGGTCTTTTAGT**

**GTTTTATAGGTTTAGGGTTTAAGGCTTAGGATTTTTTTTTTGTATTTTTTAGGATTAAGC**

**ATATACAATTTACTTCTTTTTAGGGTTTAGAGC**

**>POTJ617TR reverse BES of** 083D09

**TTGTCGGGATTTTTATTCATTTAGTAGCGTCCCTATGTTGGACACGACTTAGAGGTTATT**

**TGTATAGGTTGCTTATAACTAAATTGCTCGCTTTTAGCGATTGGATTGTTATTGTTGCCC**

**TTGGGGCTATTGTGGTTAGCTGCAGGAATGTGATCTATGCCTAGAAGGGATATGTTCTGC**

**CTACAAAACTATATGGATGCCTAAAAAGGGCTATGAGTTGCCTACGGGGATATATTGATG**

**CCTAGAAGGGCTATGAGTTGCCTACAGGGCTATATTGACGCTTAAGAAGGCTATGTGCTT**

**CCTATAGAGCCATGTTGTTGCCTAAGAGGGCTATGAGTTGTCTATAAGCTATGTTTATGC**

**CTAAGAGAGATATGTGGTGTCTACAGGGATATATTAATGCCTAAGAGGGCTATATGACTG**

**CCTACGGGACTAGGGGACTACCGATAGGGGTATATAGGTTGATTGGCACCTTTCGGGCTT**

**ATGAGGGCCTGAGTAGGTGGTCTTGTGTACTGTTTGTACCTGTCGAGCTTATGGGGGATT**

**GGTTAGGTTGTTGTTTTATTATTTTTGGATAAGTTTAGATTTAGGAGCAGGTCAGTACAC**

**TTATCTTATCCTTGATTTATTTATCAGATTATTCCACAGGATACCTCATCATAGTCTATT**

**GCCTTTCATACTCTGTACATTATTTTGTACTGACGCCCAATTGCCTTAGGGGCTCTGCAT**

**TCATGCATGCAGGTCCTGACAGACGACTGAGTAGACCTCCTCAGCAGCAGGATTGACTTT**

**TATCCGGTTGGCTAGCCCCTTTCCTCCGGAGCTACCAAAGTTGGGAGGTTTGTTACCTTT**

**TATTGTATATATCTTTA**

**>POTBD28TF forward BES of** 013E08

**AAATAGATGCGCGAAAAAATATGGAGGCAAACAATCCAACATTGCAAGAGTTCAGCATTT**

**AGTGATTTTGGACACCTTCAAGCTGCTGCATACAACCTTTCCGTATATATCGTGGCCTTT**

**GGGATGGAACAAGCTATGCACACTCATTGAAAAATGTACTCGCGATATCAAGGTCACTGC**

**TGTCCAGTGGATTAAACCACCAGCCAGGTGGTTTAAATTAAATACGGATGGGAGTGCCTT**

**GAGTAATCCGGGCAGCATAGGAGCAGGGGGTGTGCTCAGGAATTCTTTGGGAGATATTAT**

**CTTTGCATTTTCAACCCCGTTAAGCGAGGGAACCAATAACCAAGCGGAAGTGGAGGCTGC**

**TTTATTTGGTTTATCGTGGTGTGTACAATTGAATTATAAGAATGTGATCCTTGAAGTCGA**

**CTCCCAACTCCTTGTGGATTGGCTTATGAACAACAAATCAATATCGTGGTCTATCTCACC**

**CCAAATGCAACAGCTTCACCAAATTATCAATCAACTCACTCACTTCATTTGCATTCATAC**

**TCTTAGGGAAGCAAATTTTGTGGCAGACTCATTATCCAAACACAGTCACCAGCTCACCAG**

**TCCTCACGTGTACTTCAGCAGCCAGCAACTCCCCAAACTAACAGCAACGTATCTTCAACA**

**AGACC**

**>POTBD28TR reverse BES of** 013E08

**ACCTAAATCCCATTTAAACAACAAAGAAAAACTTAAAAAACATTAAAACCTAGAAAACCT**

**CTAAAAAATCTTACCCCTAACAAACTTAAACACTAAACCCTACACCCATTTTGACCCAAA**

**AAAAACTTAGGAGACCTTAAATGTTAAACTCAAAGCCTAAGAAACCTAAACCCATTTCCT**

**GAGGGATAAACCCCAAAACCTAAGCCTTATGAAACTCTAAGAAGCCCATTTTCCTAATAA**

**ATCCTAAATCCTAAGAAAAGCTAAACACTAAAAACCTTTAAAACCTAAACACTAAACCCT**

**AAATGACCCAAAAAAAAACCTAGGACCTTAAATGTTAAATTCAAATCCTAAGAAACTTAA**

**ACCCATTTCCTTAGGGATAAACCCCCAACCCTAAGCCTTATGAAACTCTAAGAAGCCCTA**

**AACCCTAAGAAAAGCTAAACACCAAAAACCTTTAAAACCCATTTACTAAAAATCCCAAAA**

**ACAAAACTTAAAAACCCTAGGAAAAGCTAAACACCAAAAACCTTTAAAACCCATTTACTT**

**AAAATCCCAAAAACAAAACTTAAAAACCCATTTAAAATTCAATAATCTAAAACCCCAAGT**

**CCTAAGCCCTTAGAGCTTATATATCGCAAAAACAATAATGATCCTAAAAAATCTTTAACA**

**ACCCATTTTTGAAAAAAACTCGTGACCGAAATTTTTTGAGAAAAACTTAAGCTCTAAACC**

**CTAACCTAAAAACCCTTAACCAAATACCTAAACTCTTCTTAATCATGTTCAGATTATATC**

**ATTATTCAATATGCTCTATTTAGTTATATATATTTGTTCAGCTTTATCATGTCA**

**>POTBU78TF forward BES of** 017N11

**ATTTTAGGTTAGGGTTTAAGGTTTAGGGCTTTGGATTTTTTTTAGGTTTAGGGATTTAGG**

**TTAATGGTTTTATCTAGGATTTTATGGTTTAGAGCTTTGGGTTTTTGAGTTAAGGTTTTT**

**ATATAAGTTCTTAGGTTCTAGGCTTATAGTTTTTTTAGCATTAGGATTTTAGGTTATGGG**

**TTTTGTCTAGATTTTTTGTGTTGCAAGATTTTAAGGTTATGGTCTTTTAGGTTTTTATAG**

**GTTTAGAATTTTGGAATTAGTTTTTTTTTGTGTTTTAGGGTTGAGCATATACGATTTAGG**

**GCTTTATAGGGCTTTTGGTTTCCTTTGGGATTTTAAGGGTTAAGTATTTGTTAGGGTTTT**

**TCAGGGTTTAGGGTTATGGGCATAGGGTATTTTCTGTTTTTTAGATTTATTGTTTTTAGA**

**TTTCTAGATTTTACAATTTAGAGATTAGGTTTTTTAGGGGTTTTAAATGGTTTTTAGGGT**

**TTATAGGATTTAGGGTTCTTAAGGTTTAGGGTTTCTTGGGGTTTTAGGTTTAGTATTTAT**

**GGTTTTAGGTTTGGGTTTAGGGTTTAGGGCTCTTTTATGGTCTAGGGTTTTATGTTAAGG**

**GTTTTGTCTAAGTTTTTAGGGTTTAGGGCTTTGGATTTTTT**

**>POTBU78TR reverse BES of** 017N11

**ATGGTGGGGGCTGCCATGCGCTAGTAGCAAATGGTTCAGCAGGCCTATAAAACATTAACC**

**TAAGAAACCCAAAGCCCATTTCCCATTTAACCTAGATAAAACCCTTAATCTAGCACACAA**

**AAACTAAAAAAATCCTGAGCCATAAA**
